# First European live birth following uterine and adnexal transposition for fertility preservation: a case report and overview of reported neonatal outcomes

**DOI:** 10.1016/j.xfre.2026.01.004

**Published:** 2026-01-30

**Authors:** Daniela Huber, Deborah Wernly

**Affiliations:** a Department of Gynecology and Obstetrics, Valais Hospital, Sion, Switzerland; b Department of Pediatrics, Gynecology and Obstetrics, Geneva University Hospitals, Geneva, Switzerland

**Keywords:** Uterine transposition, fertility preservation, rectal cancer, live birth, intrauterine growth restriction

## Abstract

**Objective:**

To report the first live birth in Europe, and the fifth worldwide, after uterine and adnexal transposition (UT) for fertility preservation in rectal cancer, and to review all published neonatal outcomes after UT.

**Design:**

Single-patient case report with comprehensive review of reported live births after UT.

**Subject:**

A 28-year-old nulliparous woman with locally advanced rectal adenocarcinoma (uT3 N1 cM0, pMMR/MSS).

**Exposure:**

Laparoscopic UT before pelvic chemoradiation, followed by uterine reimplantation during total mesorectal excision.

**Main Outcome Measures:**

Obstetric and perinatal outcomes, and placental histopathology.

**Results:**

The patient conceived naturally 18 months after uterine reimplantation. Pregnancy was complicated by intrauterine growth restriction (IUGR) and oligohydramnios, leading to elective cesarean delivery at 37 + 1 weeks of a healthy male newborn (2110 g; Apgar 9/10/10). Placental histology revealed maternal vascular malperfusion. Review of the four previously published live births confirmed natural conceptions, cesarean deliveries at 36–38 weeks, and uniformly favorable neonatal outcomes, although three pregnancies were complicated by IUGR.

**Conclusion:**

Successful pregnancy and live birth are achievable after UT and subsequent uterine reimplantation. These cumulative cases demonstrate that a temporarily displaced and later reimplanted uterus can sustain gestation to term. Given the observed risk of impaired fetal growth, enhanced third-trimester surveillance and routine placental examination are recommended. Uterine transposition represents a promising fertility-preservation option for young women requiring pelvic radiotherapy.

The incidence of early-onset colorectal cancer (CRC) is rising worldwide, particularly among women under 50 years (1, 2). Rectal tumors show the steepest rise and frequently require pelvic chemoradiation, which irreversibly damages both the ovaries and the uterus. Given that the median age at first pregnancy in Europe now exceeds 30 years (3, 4), an increasing proportion of young women may be exposed to gonadotoxic treatment before completing childbearing. Conventional fertility-preservation strategies, such as ovarian transposition or gamete and oocyte cryopreservation, preserve hormonal and genetic potential but cannot prevent radiation-induced fibrosis and vascular injury of the uterus. Consequently, uterine factor infertility often persists even when ovarian function is preserved (1, 2, 3).

Uterine transposition (UT), first described by Ribeiro et al. (4) in 2017, relocates the uterus and adnexa above the pelvic brim during radiotherapy while maintaining perfusion through the ovarian vessels. After neoadjuvant oncologic treatment, the uterus is reimplanted in its anatomic position concurrently with colorectal resection, potentially allowing for future gestation. Since its introduction, UT has evolved into a feasible fertility-preservation option for rectal, pelvic sarcoma, and gynecologic malignancies (5, 6, 7, 8). To date, only four live births (3 in Brazil and 1 in Peru) have been reported after successful uterine reimplantation (9, 10, 11, 12).

We describe the fifth live birth worldwide and the first in Europe after UT, occurring in a patient treated for locally advanced rectal cancer. Although the surgical technique has been previously described (13), the present manuscript not only confirms reproductive feasibility, but provides detailed obstetric and neonatal outcomes, and the first placental histopathological assessment after this technique, revealing maternal vascular malperfusion associated with intrauterine growth restriction (IUGR) and oligohydramnios likely related to impaired uterine perfusion after reimplantation.

## Case presentation

A 28-year-old nulliparous woman was diagnosed in June 2022 with moderately differentiated low rectal adenocarcinoma, at 7 cm from the anal verge (uT3 N1 cM0, pMMR/MSS). Neoadjuvant treatment followed the PRODIGE 23 protocol, beginning with six cycles of induction FOLFIRINOX. This was followed by long-course chemoradiotherapy: a total dose of 50.4 Gy was delivered (45 Gy pelvic field with a 5.4 Gy tumor boost in 1.8 Gy fractions) using cone-beam computed tomography (CT) guidance, alongside concomitant oral Capecitabine (825 mg/m^2^ BID, 5 d/wk). The oncologic management and surgical technique for UT applied in this case have been previously published in detail (13).

**Figure 1:**
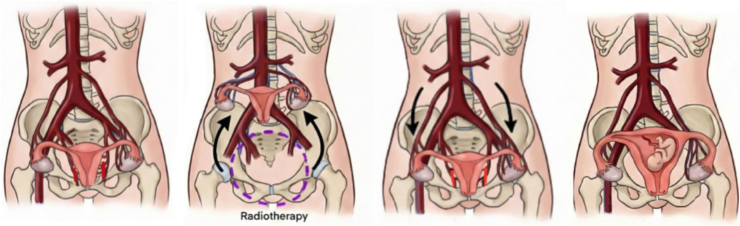
Schematic of uterine and adnexal transposition and reimplantation. (**A**) Pre-operative state: Identification of the rectal tumor and initial surgical planning. (**B**) Transposition phase: The uterus and adnexa are mobilized, the uterine arteries are divided, and the organ is cranially transposed (∼22 cm) to the upper abdomen, anchored outside the planned pelvic radiation field. (**C**) Reimplantation phase: After the completion of chemoradiation, the uterus is returned to the pelvis and reanastomosed to the vaginal cuff during the total mesorectal excision. (**D**) Reproductive outcome: Successful spontaneous conception and subsequent cesarean delivery.

During the procedure, both uterine arteries were divided close to the cervix, and uterine perfusion was maintained through the ovarian vessels. Incidentally, stage II endometriosis involving the left uterosacral ligament was identified and excised. The vagina was transected at the cervicovaginal junction, and the uterus and adnexa were transposed to the upper abdomen and anchored to the anterior abdominal wall (13), with an estimated cranial displacement of the uterus above the pelvic brim of approximately 22 cm (Fig. 1). No cervico-umbilical anastomosis was created. Monthly gonadotropin-releasing hormone (GnRH) agonist injections (Pamorelin 3.75 mg) were administered throughout chemotherapy and until uterovaginal reimplantation, preventing menstruation during displacement. Normal menses returned 2 months after reimplantation.

**Figure 2:**
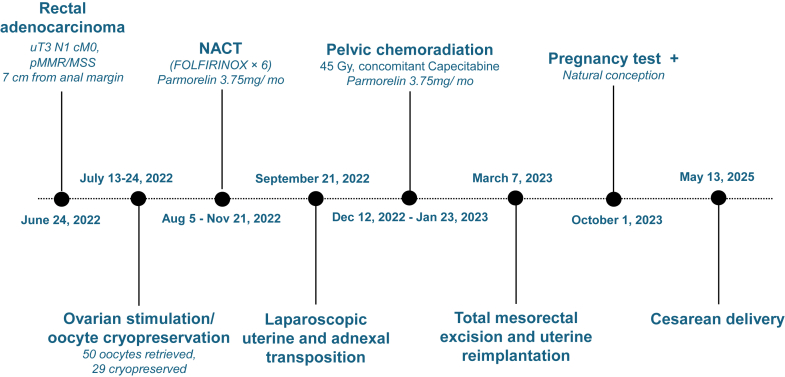
Chronological timeline summarizing oncologic, surgical, and obstetric milestones from diagnosis to delivery.

After chemoradiation, total mesorectal excision with uterine reimplantation and cervicovaginal anastomosis was performed. Pathology confirmed a complete pathologic response (ypT0 N0 R0). The patient remained disease-free through 2024. The chronological timeline of all oncologic, surgical, and reproductive events is illustrated in Figure 2.

A cranial vaginal stenosis developed after reimplantation, likely secondary to radiation-induced fibrosis of the upper vaginal segment. Similar cases have been reported (14). Pelvic-floor physiotherapy, including manual scar mobilization and relaxation exercises, restored vaginal patency over 8 weeks, with only a minimal asymptomatic residual stenosis.

Nineteen months after reimplantation, conception occurred naturally without ovulation induction or assisted reproductive techniques. Fetal growth was normal until 32 weeks + 4 days, with an estimated fetal weight at the 40th percentile and normal amniotic fluid volume. At 36 weeks + 4 days, ultrasound revealed new-onset IUGR (estimated fetal weight at the 10th percentile) associated with oligohydramnios (amniotic fluid index = 5 cm). Elective cesarean section at 37 weeks + 1 day (May 13, 2025) yielded a male neonate weighing 2110 g (<3rd percentile), with Apgar scores of 9/10/10. No pelvic adhesions were observed intraoperatively.

**Figure 3:**
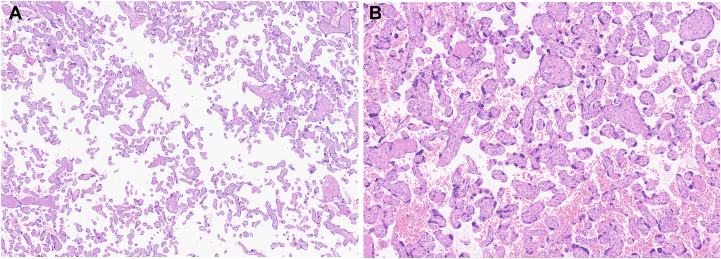
(**A**) Representative hematoxylin–eosin section of the placenta showing distal villous hypoplasia (4×), consistent with maternal vascular malperfusion. (**B**) Representative hematoxylin–eosin section of the placenta showing increased syncytial knotting (10×), consistent with maternal vascular malperfusion.

The placenta was hypotrophic (270 g, <3rd percentile) and exhibited accelerated villous maturation and increased syncytial knotting, consistent with maternal vascular malperfusion. (Fig. 3A and B). Both mother and child were healthy at the 8-month follow-up. Written informed consent for publication of all clinical details and images was obtained from the patient.

We searched MEDLINE (PubMed), Embase, Scopus, and Web of Science for reports of live births after UT from July 1, 2023 (the date of the first reported live birth after UT by Ribeiro et al. [4]) through October 20, 2025. Search terms combined: (“uterine transposition” OR “uterus transposition” OR “uterine relocation” OR “uterine transposition” OR “UOT” OR “utero-ovarian transposition”) AND (pregnancy OR “live birth” OR delivery OR childbirth). We limited to human studies and screened titles/abstracts, then full texts, to include case reports/series or observational studies documenting a live birth after UT (with or without prior pelvic radiotherapy). We excluded studies on ovarian transposition alone, editorials without patient and obstetrics outcomes, and nonhuman studies. Reference lists of eligible articles and forward citations were hand-searched to identify additional reports. Key anchor references included the first live birth after UT (9) and subsequent narrative/systematic overviews summarizing UT pregnancy outcomes. Two reviewers screened records independently. The PRISMA diagram is presented in Supplemental Figure 1 (available online) [15].

## Discussion

Young-onset CRC frequently presents with aggressive features and advanced staging, often necessitating multimodal neoadjuvant chemoradiation. Although lifesaving, pelvic irradiation poses a dual threat to reproductive potential: it carries a high risk of permanent primary ovarian insufficiency and inflicts significant “uterine factor” infertility. Radiation-induced damage to the uterus includes reduced volume, impaired blood flow, and irreversible myometrial fibrosis and endometrial atrophy, which collectively compromise pregnancy maintenance and obstetric safety, even when ovarian function is surgically or pharmacologically preserved.

The reproductive prognosis for women receiving standard multimodal pelvic radiotherapy for rectal cancer is exceptionally poor. Although a few pregnancies have been reported after pelvic irradiation for anal or rectal cancer, including a vaginal delivery after a mean uterine dose of 34.5 Gy (16) and a twin pregnancy after intensity-modulated radiotherapy that limited the uterine body dose to 16 Gy (14), these cases are clinical rarities.

Protecting the uterus during radiotherapy remains a critical challenge in survivorship care. Although conventional strategies such as oocyte or embryo cryopreservation should be performed before treatment, they do not address the loss of a functional uterus. Uterine and adnexal transposition offers a promising solution by temporarily relocating the organ to the upper abdomen, effectively placing it outside the ionizing radiation field while maintaining essential perfusion via the ovarian vessels.

Preservation of the uterine arteries during pelvic surgery has traditionally been regarded as essential for subsequent fertility because of their major contribution to uterine perfusion and endometrial receptivity. In the context of UT, however, division of these arteries is unavoidable to allow displacement of the uterus and adnexa outside the radiation field. Uterine perfusion is therefore maintained through the ovarian arteries and their utero-ovarian anastomoses.

Despite reports of frequent postoperative uterine-artery occlusion after radical trachelectomy (87%–88%) (17) or intentional arterial division in certain surgical techniques, published cohorts have not demonstrated a consistent difference in pregnancy rates attributable to uterine artery patency. Instead, conception success appears to depend primarily on the surgical approach (vaginal vs. abdominal), residual cervical length, postoperative adhesions, complications such as uterine or ovarian necrosis and infections, and the use of assisted reproduction techniques (18).

Evidence from benign gynecology supports the physiological tolerance to uterine artery interruption: in a randomized controlled trial with 2-year follow-up, Streuli et al. (19) confirmed unchanged serum antimüllerian hormone (AMH) and antral follicle count after laparoscopic myomectomy with definitive bilateral uterine artery occlusion. These findings support the concept that ovarian-based collateral flow is sufficient to sustain endocrine and reproductive activity, although it may limit uterine vascular remodeling during pregnancy.

Across the four previously published UT pregnancies and our case, neonatal outcomes were overall favorable (9, 10, 11, 12) (Table 1). However, IUGR occurred in three pregnancies [10, 14], one associated with maternal hypertension. These observations provide important insights into the gestational potential and obstetric safety of a uterus temporarily displaced outside the pelvis and later reimplanted, confirming that successful pregnancy and delivery are possible even after bilateral uterine-artery division. Our case provides the first histopathologic evidence of placental maternal vascular malperfusion, supporting the hypothesis of impaired uteroplacental remodeling after reimplantation. Similar vascular abnormalities have been reported after uterus transplantation (20), suggesting shared mechanisms of adaptive revascularization. Conventional fertility-preservation strategies (e.g., oocyte/embryo cryopreservation) should be offered alongside UT, particularly given the theoretical risk of thrombosis or ischemia of the transposed uterus.

**Table. 1:** Obstetric and perinatal outcomes of reported live births after uterine transposition.

Study	PMID	Indication	GA at delivery	Birth weight	Perinatal findings
Ribeiro et al. 2023 (9)	36863432	Low-grade myxoid liposarcoma	36 wk + 2	2,686	Normal
Lopez et al. 2023 (10)	37549972	Rectal adenocarcinoma	36 wk	2,500	IUGR, oligohydramnios
Ribeiro et al. 2023 (12)	37898483	Rectal adenocarcinoma	38 wk	NR	NR
Moretti-Marques et al., 2024 (11)	39138911	Cervical adenocarcinoma	36 wk + 3	2,255	IUGR, maternal hypertension
Present case, 2025		Rectal adenocarcinoma	37 wk + 1	2,110	IUGR, oligohydramnios

*Note:* Birth weights are expressed in grams. GA = gestational age; IUGR = intrauterine growth restriction; NR = not reported; PMID = PubMed IDentifier.

All reported live births resulted from spontaneous conception. Assisted reproduction may be considered after 6–24 months of unsuccessful attempts, guided by patient preference, fertility assessment, and oncologic safety. Consistent with radical trachelectomy literature (21, 22, 23, 24), cesarean delivery remains the safest mode of birth after uterine reimplantation, minimizing mechanical stress on the cervicovaginal anastomosis. Furthermore, prior pelvic radiotherapy may induce fibrosis and decrease the elasticity and compliance of the pelvic floor and vaginal canal, thereby contraindicating vaginal delivery. To date, no cases of uterine rupture or other maternal morbidity related to UT during pregnancy have been reported.

Pregnancies after UT should be considered high risk. Enhanced third-trimester fetal-growth surveillance and Doppler velocimetry may enable early detection of growth impairment. In the absence of other obstetric complications, elective cesarean delivery between 36 and 38 weeks should be planned to minimize the risk of anastomotic rupture and pelvic floor trauma, consistent with the timing routinely adopted after radical trachelectomy.

To date, no second pregnancies have been reported after UT. In the absence of a genetic condition requiring a delayed hysterectomy, such as Lynch or Li-Fraumeni syndrome, subsequent gestation appears theoretically feasible.

## Conclusion

Pregnancy and live birth are achievable after a UT and subsequent uterine reimplantation. Among the five live births reported to date, three pregnancies were complicated by IUGR, including the present case, underscoring the need for enhanced third-trimester fetal growth surveillance and systematic placental examination in future pregnancies. Conventional fertility-preservation options should continue to be offered alongside UT to ensure comprehensive reproductive safety. Collectively, these five cases—including this first European-reported live birth—demonstrate that a reimplanted uterus can sustain gestation to term, representing a significant advance in fertility preservation for women requiring pelvic radiotherapy. Future multicenter registries are warranted to define obstetric surveillance protocols and to assess the long-term reproductive potential after UT.

### Declaration of Interests

D.H. has nothing to disclose. D.W. has nothing to disclose.

## CRediT Authorship Contribution Statement

**Daniela Huber:** Writing – review & editing, Writing – original draft, Validation, Supervision, Project administration, Methodology, Data curation, Conceptualization. **Deborah Wernly:** Writing – review & editing, Formal analysis.

## References

[1] Lohynska R., Jirkovska M., Novakova-Jiresova A., Mazana E., Vambersky K., Veselsky T. (2021). Radiotherapy dose limit for uterus fertility sparing in curative chemoradiotherapy for rectal cancer. Biomed Pap.

[2] van der Kooi A.-L.L.F., van Dorp W. (2025). The impact of radiotherapy on the uterus and its implications for pregnancy. Semin Reprod Med.

[3] Rozen G., Rogers P., Chander S., Anderson R., McNally O., Umstad M. (2020). Clinical summary guide: reproduction in women with previous abdominopelvic radiotherapy or total body irradiation. Hum Reprod Open.

[4] Ribeiro R., Rebolho J.C., Tsumanuma F.K., Brandalize G.G., Trippia C.H., Saab K.A. (2017). Uterine transposition: technique and a case report. Fertil Steril.

[5] Baiocchi G., Vieira M., Moretti-Marques R., Mantoan H., Faloppa C., Damasceno R.C.F. (2021). Uterine transposition for gynecological cancers. Int J Gynecol Cancer.

[6] Leitao M. (2023). Uterine transposition: a simple yet revolutionary means of fertility preservation for women with cancer. Int J Gynecol Cancer.

[7] Ribeiro R., Baiocchi G., Tsunoda A.T., Linhares J.C., Pareja R. (2019). Uterine transposition technique: update and review. Minerva Ginecol.

[8] Apelian S., Vest A., Yasukawa M., Wellcome J., Kohut A., Imudia A.N. (2025). Uterine transposition for fertility preservation and ovarian conservation in patients undergoing pelvic radiotherapy. Obstet Gynecol.

[9] Ribeiro R., Anselmi M.C., Schneider G.A., Rodrigues Furtado J.P., Mohamed Abau Shwareb M.G., Linhares J.C. (2023). First live birth after uterine transposition. Fertil Steril.

[10] Lopez A., Perez Villena J.F., Guevara Jabiles A., Davila K., Sernaque Quintana R., Ribeiro R. (2023). Uterine transposition and successful pregnancy in a patient with rectal cancer. Int J Gynecol Cancer.

[11] Moretti-Marques R., Franca I.B., De Cillo P.E., Alvarenga-Bezerra V., Helito J.K., Filho D.C. (2024). First birth after uterine transposition in low-volume lymph node metastasis of cervical cancer: a long journey for success. J Surg Oncol.

[12] Ribeiro R., Baiocchi G., Moretti-Marques R., Linhares J.C., Costa C.N., Pareja R. (2023). Uterine transposition for fertility and ovarian function preservation after radiotherapy. Int J Gynecol Cancer.

[13] Huber D., Simonson C., Fournier I., Dischl-Antonioni I., Pena Rios F.J., Francey I. (2024). Utero-ovarian transposition before pelvic radiation in a patient with rectal cancer: a case report and systemic literature review. Front Surg.

[14] Lohynska R., Jirkovska M., Novakova-Jiresova A., Mazana E., Vambersky K., Veselsky T. (2021). Radiotherapy dose limit for uterus fertility sparing in curative chemoradiotherapy for rectal cancer. Biomed Pap Med Fac Univ Palacky Olomouc Czech Repub.

[15] Page M.J., McKenzie J.E., Bossuyt P.M., Boutron I., Hoffmann T.C., Mulrow C.D. (2021). The PRISMA 2020 statement: an updated guideline for reporting systematic reviews. BMJ.

[16] Hürmüz P., Sebag-Montefiore D., Byrne P., Cooper R. (2012). Successful spontaneous pregnancy after pelvic chemoradiotherapy for anal cancer. Clin Oncol (R Coll Radiol).

[17] Tang J., Li J., Wang S., Zhang D., Wu X. (2014). On what scale does it benefit the patients if uterine arteries were preserved during ART?. Gynecol Oncol.

[18] Kohler C., Plaikner A., Siegler K., Hertel H., Hasenbein K., Petzel A. (2024). Radical vaginal trachelectomy: long-term oncologic and fertility outcomes in patients with early cervical cancer. Int J Gynecol Cancer Off J Int Gynecol Cancer Soc.

[19] Streuli I., Ramyead L., Silvestrini N., Petignat P., Dubuisson J. (2025). Impact of definitive uterine artery occlusion on ovarian reserve markers in laparoscopic myomectomy: a randomized controlled trial with 2-year follow-up. Hum Reprod.

[20] Brännström M., Bokström H., Hagberg H., Carlsson Y. (2025). Maternal and perinatal outcomes of live births after uterus transplantation: a systematic review. Acta Obstet Gynecol Scand.

[21] Takada S., Ishioka S., Endo T., Baba T., Morishita M., Akashi Y. (2013). Difficulty in the management of pregnancy after vaginal radical trachelectomy. Int J Clin Oncol.

[22] Knight L.J., Acheson N., Kay T.A., Renninson J.N., Shepherd J.H., Taylor M.J.O. (2010). Obstetric management following fertility-sparing radical vaginal trachelectomy for cervical cancer. J Obstet Gynaecol.

[23] Ma L.-K., Cao D.-Y., Yang J.-X., Liu J.-T., Shen K., Lang J.-H. (2014). Pregnancy outcome and obstetric management after vaginal radical trachelectomy. Eur Rev Med Pharmacol Sci.

[24] Johansen G., Lönnerfors C., Falconer H., Persson J. (2016). Reproductive and oncologic outcome following robot-assisted laparoscopic radical trachelectomy for early stage cervical cancer. Gynecol Oncol.

