# Supplementary figures and images for: First European live birth following uterine and adnexal transposition for fertility preservation: a case report and overview of reported neonatal outcomes
Source: F S Rep. 2026 Jan 30;7(2):120–4. doi: 10.1016/j.xfre.2026.01.004 (PMC13100844; doi:10.1016/j.xfre.2026.01.004)

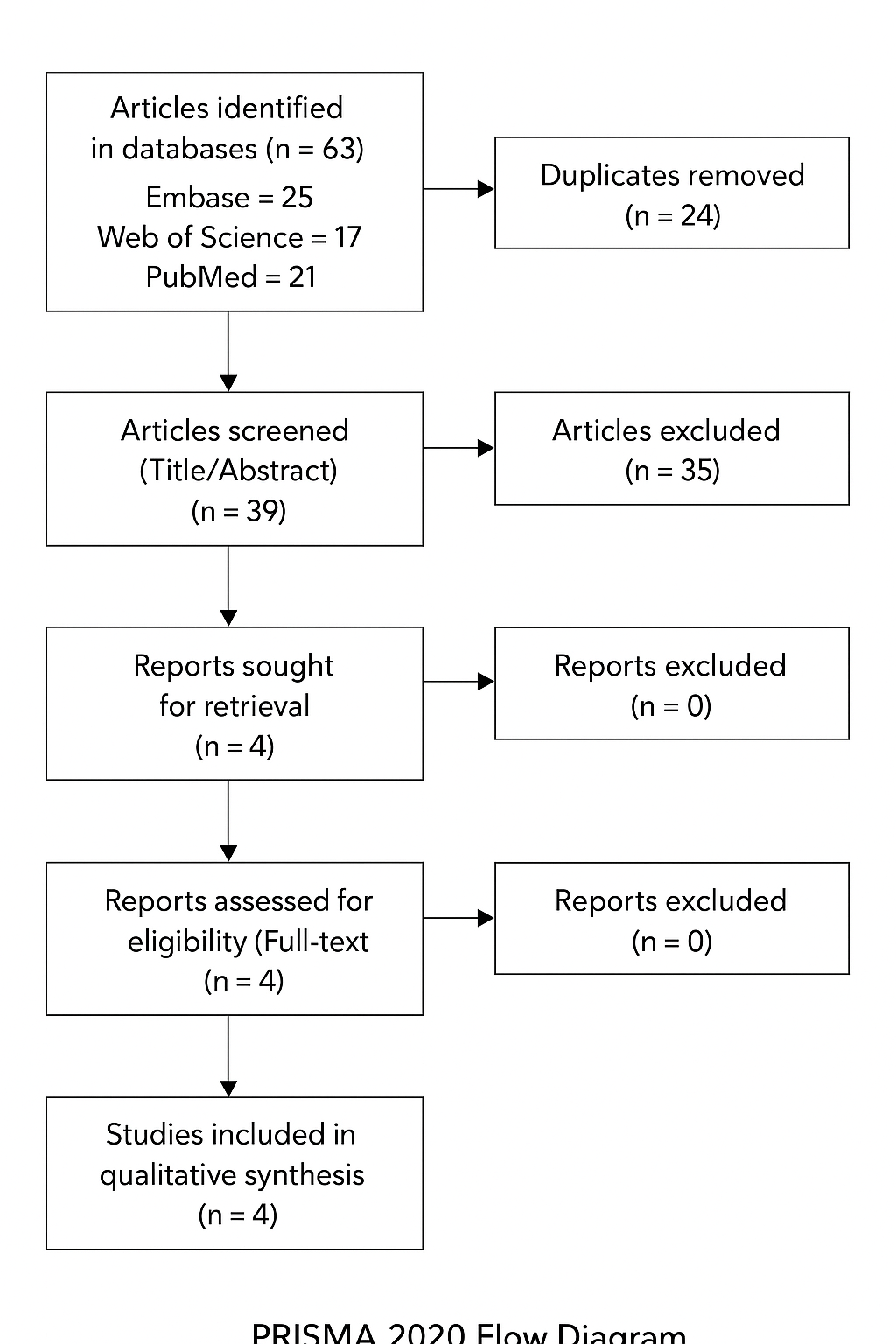


Figure 1. Prisma flow diagram (16)

Supplement: Supplemental Figure 1 [file mmc1.docx]
